# Electric discharge evidence found in a new class of material in the Chicxulub ejecta

**DOI:** 10.1038/s41598-020-65974-2

**Published:** 2020-06-03

**Authors:** Gunther Kletetschka, Adriana Ocampo Uria, Vojtech Zila, Tiiu Elbra

**Affiliations:** 1 Institute of Geology, Czech Academy of Sciences, Rozvojová 269, Prague 6, 16500 Czech Republic; 2 Department of Applied Geophysics, Charles University, Albertov 6, Prague 2, 12843 Czech Republic; 3 Geophysical Institute, University of Alaska, Fairbanks, 903 N Koyukuk Drive, Fairbanks, AK USA; 4 NASA Headquarters, Washington DC, 20546 USA; 5 Department of Infectious Diseases, Virology, University of Heidelberg, Heidelberg, Germany

**Keywords:** Natural hazards, Planetary science, Space physics, Geology, Geomagnetism, Geophysics, Palaeomagnetism

## Abstract

Chicxulub impact (66 Ma) event resulted in deposition of spheroids and melt glass, followed by deposition of diamectite and carbonate ejecta represented by large polished striated rounded pebbles and cobbles, henceforth, called Albion Formation^1^ Pook’s Pebbles, name given from the first site identified in central Belize, Cayo District. Here we report that magnetic analysis of the Pook’s Pebbles samples revealed unique electric discharge signatures. Sectioning of Pook’s Pebbles from the Chicxulub ejecta from the Albion Formation at Belize showed that different parts of Pook’s Pebbles had not only contrasting magnetization directions, but also sharply different level of magnetizations. Such behavior is indicative of electric discharge taking place sometimes during the formation of the Chicxulub ejecta blanket. In addition, some of the Pook’s Pebbles’ surface had recrystallized down to 0.2 mm depth. This is evidence of localized extreme pressures and temperatures during the fluidized ejecta formation which was imprinted in the outer layer of Pook’s Pebbles. Recrystallization caused formation of nanophase iron along the surface, which was revealed by mapping of both natural remanent magnetization and of saturation remanence magnetization signatures. While the spheroids’ magnetization orientation is consistent with reversed magnetic field at the time of impact, the study of the Pook’s Pebbles provided, in addition, new evidence of electric charging during the vapor plume cloud processes.

## Introduction

**Figure 1:**
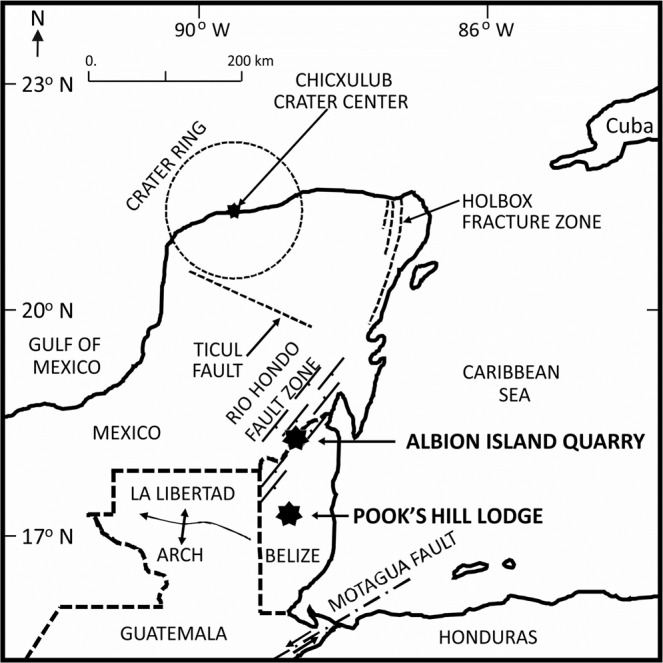
Location map of the sample collection sites. One sampling site was ~½ km in diameter quarry area, called Albion Island Quarry (latitude 18° 7′45.00″N, longitude 88°41′10.00″) and Pook’s pebbles were collected from the site near Pook’s Hill Lodge (stratotype), in respect to simplified geology and geography. Chicxulub crater is outlined by 190 km dashed circle labeled “Crater ring” based on gravity anomalies^12^ and satellite altimetry data. The map contains two types of dashed lines. Thicker dashed line indicates borders between countries. Thinner dashed lines show the crater ring and major fault lines. Solid bold line shows the ocean/land border. Map was drawn using Microsoft PowerPoint for Mac, 2018, version 16.16.20 (https://www.microsoft.com/).

**Figure 2:**
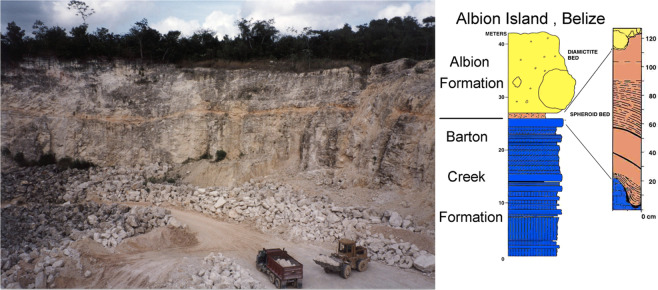
Stratigraphy documented with photograph taken by Adriana Ocampo Uria, in the Albion Island Quarry, Belize type locality for the Albion Formation. Photograph shows contrast between the units and truck for scale. Note the stratigraphic bar in the vertical profile in meters. This scale indicates an approximate 1 m thickness of the spheroid bed (shown as a light orange ribbon in the photo overlaying the Barton Creek Dolomite). The photo shows the vegetation along the skyline in the actively worked Albion Island Quarry. The stratigraphic top of the Albion Island Formation Diamectite Bed is not found at this location.

The Chicxulub impact event, from the Cretaceous-Paleogene (K-Pg) boundary, is linked to a carbonaceous chondrite meteorite collision^2^ into a volatile-rich carbonate substrate with an underlying crystalline basement in the Yucatán region, Mexico. The impact produced a globally distributed ejecta layer, and it ranks as the primary cause of this major mass extinction event, which resulted in resetting preexisting ecosystems^3^. Exposures of K-Pg boundary impact ejecta deposits were found in Belize^3,4^. In northern Belize the impact event created a spectacular outcropping of continuous ejecta blanket in the type site named as Albion Formation^5,6^. The Albion Formation ejecta blanket contained two lithostratigraphic subunits: The Spheroid Bed, and Diamictite Bed, which overlie the Cretaceous Barton Creek Dolomite. In Northern Belize, there are also present discontinuous ballistic ejecta deposits. The latter are found in abundance southeast of the Chicxulub impact crater center, approximately ~475 km, in the Cayo District of Central Belize (Fig. 1). These ballistic ejecta deposits form a 10 to 30 m-thick carbonaceous rich deposits of pebbles, cobbles, and boulders, supported by a matrix of red clay and silt (Fig. 2) with signs of thermal metamorphism (Fig. S1). This discontinuous sheet of debris rests directly on the terminal Cretaceous land surface^7^ in similar way as Albion Formation’s Diamictite that overlies an irregular karst surface of the Upper Cretaceous Barton Creek Formation along the northern flanks of the Maya Mountains^7^.

The composition and stratigraphy of the ejecta in the southern Yucatan Peninsula suggest that three processes were involved during the ejecta deposition at different crater radii from the center^7^. The earliest process involves the rapid expansion of a volatile-rich cloud producing a deposit of relatively uniform thickness (Spheroid Bed). The second emplacement process also involves a volatile-rich cloud, but with less velocity and with greater mass density traveling as a debris flow, and a larger range of deposited grain size (Albion Formation Diamictite Bed)^1^. Beyond a radius of ~360 km (or about four crater radii) the deposition changed into coarse, sometimes well-polished and striated pebbles and cobbles forming the proposed name of Albion Formation Pook’s Pebble Bed, being the type locality the Pook’s Hill Lodge in Teakettle Belize. This locality was first identified in the Cayo District of Central Belize^1^ and it was proposed that the process that deposited the Pook’s Pebble Bed involved fluidized ejecta with contrasting velocities (hot fine material moving pass the coarser objects with high-velocity resulting in the collisions and heat transfer into the coarser objects, and producing plastic deformation). The depositional emplacement processes relate to three ejecta transport regimes. The low energy part of the condensing impact vapor plume (mixture of carbonates and water vapor) as it expanded beyond the advancing ejecta curtain entrained small clasts of the Albion Formation Spheroid Bed. The ejecta curtain material was transformed by heat due to interaction with the hot condensing gases and transported as debris flow to form the Albion Formation Diamictite Bed. High-velocity abrasion of large limestone/dolostone fragments, at relatively lower temperature than Diamictite Bed, resulted in deposition of the Pook’s Pebble Bed.

## Materials and Methods

### Spheroid material

We collected hundreds of samples from three distinct carbonate sections (Spheroid Bed, Diamictite Bed, and Pook’s Pebble Bed) that were part of the Albion Formation unit: The lowest unit is 1 to 2 m thick spheroid bed that follows the preexisting karst topography and contains abundant accretionary lapilli. The next unit deposited was 8–15 m thick Diamectite bed material that includes matrix supported clasts of angular to sub rounded shape, some of them with well-preserved striated surfaces. This unit contains clasts over 5 m in size with accretionary rinds (Fig. S2). In central Belize, the Diamectite Bed is missing, however, and instead, directly overlaying the Spheroid Bed is the Pook’s Pebble bed^8^. The Albion Formation Pook’s Pebble Bed is about 4 m thick, and is a matrix- and clast-supported sub-to well-rounded pebbles and cobbles. More than 50% of these carbonaceous pebbles have distinctive pits ranging from 1 mm to 1–2 cm in size. Portions of these pebbles appear to have smooth polish with defined lineation that grade into striations (Figs. S3–S6). The Pook’s Pebble carbonate comes from early Maastrichtian due to common occurrence of fossil foraminifera^8,9^. The Pook’s Pebbles are made of pink to white microcrystalline limestone sometimes with microfossils and often contains cherty veins and nodules of microspherulitic nature. These nodules commonly have high relief and a rough bleached surface that is surrounded by polished and striated surface (Fig. S4). The microfossils found in these pebbles are similar to the microfossils of the Yucatan platform target rocks^10^.

**Figure 3:**
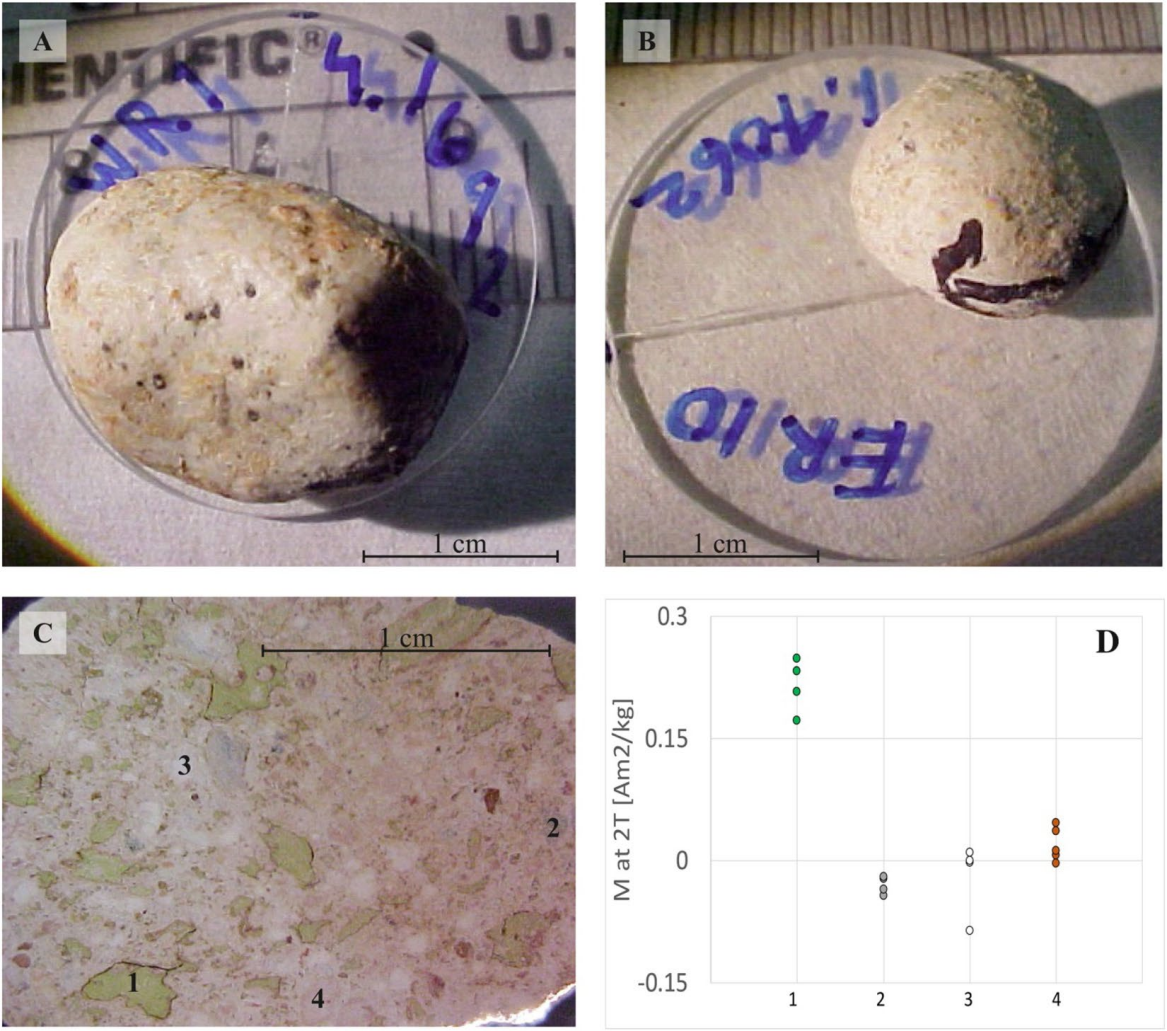
Albion Formation Spheroid Bed samples: (**A**,**B**) Photographs of two oriented samples of spheroid nodules from the Spheroid Bed, Central Belize. The photos background scale is in millimeters, the blue label indicates sample ID and its weight in grams. 1-inch round glass slides were holders for magnetic measurements. Black marking indicates northern and horizontal direction at the time of collection from the outcrop. The dark spots on the surface are occasional fossils and chert fragments that are within the fine solidified dusty matrix of the spheroids. (photo by G. Kletetschka). (**C**) The surface of the consolidated fragment of the Spheroid Bed unit (cut smooth with the thin diamond saw) is shown in reflected light. Morphology contains the main four-unit lithologies shown by numbers 1= Devitrified impact glass (green blebs), 2 = Limestone fragments, 3 = White Spheroids, and 4 = Pink Spheroids. (photo by G. Kletetschka). (**D**) Range of magnetization of the Diamectite Bed sample identified by numbers in C.

The Albion Formation Spheroid Bed is material that contains clay spheroids (palagonite) representing devitrification products from the impact glass that formed during the K-Pg Chicxulub impact crater formation^11^. Dolomite-containing spheroid are likely altered impact-derived accretionary lapilli^5^ from the same event. The distance of the material collection site is little more than three Chicxulub crater’s radii from the crater center (Fig. 1). When one of the consolidated spheroid bed fragments is cut clean, it reveals four major components: devitrified glass, white spheroids, pink spheroids, and limestone/dolomite fragments (Fig. 2, Fig. 3C). We analyzed 19 oriented spheroids (1–3 cm in diameter) from the Spheroid Bed in Albion Island Quarry for directional magnetic analyses (see example of two spheroids’ photographs in Fig. 3A,B).

### Spheroids methods

The four types of material of the Albion Formation Spheroid Bed are; green devitrified glass, limestone/dolomite fragments, pink and white spheroids. These were extracted from the Spheroid Bed and each exposed to 2 T magnetic field while measuring their magnetic moment inside the bipolar vibrating sample magnetometer (VSM, LakeShore *model 7300, at GSFC/NASA*) (Fig. 3D). We analyzed 19 spheroids samples (collected oriented from Spheroid Bed) measurements of magnetic remanence and demagnetization by alternating magnetic field by using rock superconducting magnetometer system, (SRM, Superconducting Technology, 2 G Inc), in a vertical configuration. Both, SRM instrument system and VSM, were located in NASA’s Goddard Space Flight Center, Greenbelt, MD, USA.

### Pook’s pebbles material

The Albion Formation Pook’s Pebble Bed is material that is composed of sub and well-rounded, polished, pitted, plastically deformed and striated pebbles and cobbles in a clay rich matrix with degraded impact glass green spherules. We analyzed 20 pebbles with polish and/or striations with clast diameters ranging from 4 to 25 cm (mean diameter of 8.6 cm). The Pook’s Pebbles carbonates come both in white and pink colors, some with microfossils. Nearly all (90%) Pook’s Pebbles were fine (~5 µm) grained pink recrystallized limestones with annealed carbonate crust shown by its polish (Fig. S1). The pink color was not uniform and when examined in thin sections it’s patchy due to halos around small (~100 µm) hematite grains, indicating reduction/oxidation processes. Hematite and goethite presence have been identified in spheroids bed^13^ and thus it is likely that they contribute to the magnetization of this carbonaceous material. Oxygen-deficient water moving through the pores dissolved and re-precipitated nano-sized iron oxides around the preexisting iron oxide grains. These pebbles were abundant near the type locality Pook’s Hill Lodge in central Belize (Fig. 1). Pook’s Pebbles are mostly flatten ellipsoids (68%), but also rod (15%) and spheroidal (18%) shapes are common^14^. All seem faceted. These facets, contain both interior and exterior rounded corners. While majority (80%) samples have pits, several (35%) have pitting that covers half of the clast^14^. Pits are elliptical to circular depressions with abrupt rims. The plastically deformed larger pits look like thumb impressions made in soft clay^14^. Typical pits are 0.3–2.0 cm in diameter and are about half as deep as they are wide. In a few examples the pits are much deeper, forming 0.5–2 cm deep holes that are 0.1–0.5 cm in diameter^14^. All Pook’s Pebbles have patches of a white, chalky calcite crust that covers on average 24% of the clast. The chalky crust is a few mm thick and composed of mostly 40-µm sized calcite crystals^14^. The crust also contains about 1% sand and pebble-sized clasts (same lithology as Pook’s Pebbles), which are abundant in a few (8%) samples. Patches of the crust are a gray, translucent, coarse (100 µm-sized) calcite^14^. A few (8%) examples of this denser crust contain vugs 1–3 mm in diameter and laminations with truncations. Nearly half (43%) of the examined clasts have one facet (and only one) that is mostly covered with crust. Many (42%) of these clasts with crust on one side have extensive pitting on the opposite side. Where the crust is missing, the clast surface is typically polished and/or striated and it is apparent that the crust has been stripped away in the process. A fine polish occurs in patches and rarely covers the entire clasts. In several (~15%) examples, the harder gray translucent crust is also polished, including the small fragments that appear embedded in the crust. Striations are common, occurring in parallel sets and multiple directions, typically within distinct 2–10 mm wide gouges. These gouges also occur in parallel sets, resulting in a striated surface. Many examples of striated gouges have curves and kinks, or end in an abrupt, angular facet.

### Pook’s pebbles methods

We prepared a thin section from one of the Pook’s Pebble carbonate clasts (verified by reaction with hydrochloric acid). Then used a non-magnetic saw to cut one larger (~15 cm in length; 1.5 kg) representative Pook’s Pebble (Fig. 6) into three segments (Fig. 6A, inset), where the middle segment was 1 cm thick slab (Fig. 6A, inset). The pebble was oriented during the cutting (the long cut was in vertical plane, 11 degrees East from North so that the cut fragments were similar oriented in respect to the original pebble orientation mark). Specifically, the arrow marking on the pebble indicated the strike and dip of the marked surface (N11E,90) and the resulting sub-fragments contain the same orientation direction (green arrows to the left on Fig. 6B on each sub sample). This slab was then cut into 52 individual cubes, each of them 1 cm in size (Fig. 6B, inset). We obtained paleomagnetic direction for each sample (Fig. 6C) along with the NRM intensity (Fig. 6D), and magnetization of saturation remanence (Msr) (Fig. 6E).

We obtained two cylindrical core samples from Pooks Pebble 04 (Fig. S5) using a non-magnetic drill. Each of the two cores: PP01, PP02, 1 inch in diameter, was split in to two pair samples (pair PP01A, PP01B and pair PP02A, PP02B). Two cylindrical cores were measured using SRM instrument for NRM, Msr and also thermal remanent magnetization (TRM) with application of Schoensted paleomagnetic oven at NASA, GSFC. TRM was acquired at ambient field of 30 µT and then the samples were magnetically saturated to obtain Msr after heating, using the VSM electromagnet.

## Results

We measured saturation magnetization of four main components of the Albion Formation Spheroid Bed: green devitrified glass spherules, limestone fragments, white spheroids, and pink spheroids. This was done in order to check if, magnetically, these ejecta materials differ and the magnetic properties could be used as a unique identifier for ejecta blanket deposits. These measurements show that this technique could be used as a signature for impact ejecta deposits detection by planetary rovers^15^. The highest magnetization level (0.175–0.252 Am^2^/kg) at 2 T external magnetic field was obtained from the green devitrified impact glass. Other three components overlapped with each other, however they had clearly lower magnetization than the green devitrified impact glass spherules. The magnetization of limestone/dolomite fragments ranged between −0.052 and −0.017 Am^2^/kg, while carbonate spheroids had a larger spread – between −0.085 and 0.015 Am^2^/kg. The pink carbonate Pook’s pebbles Bed spheroids had slightly higher magnetization level – between 0 and 0.051 Am^2^/kg.

**Figure 4:**
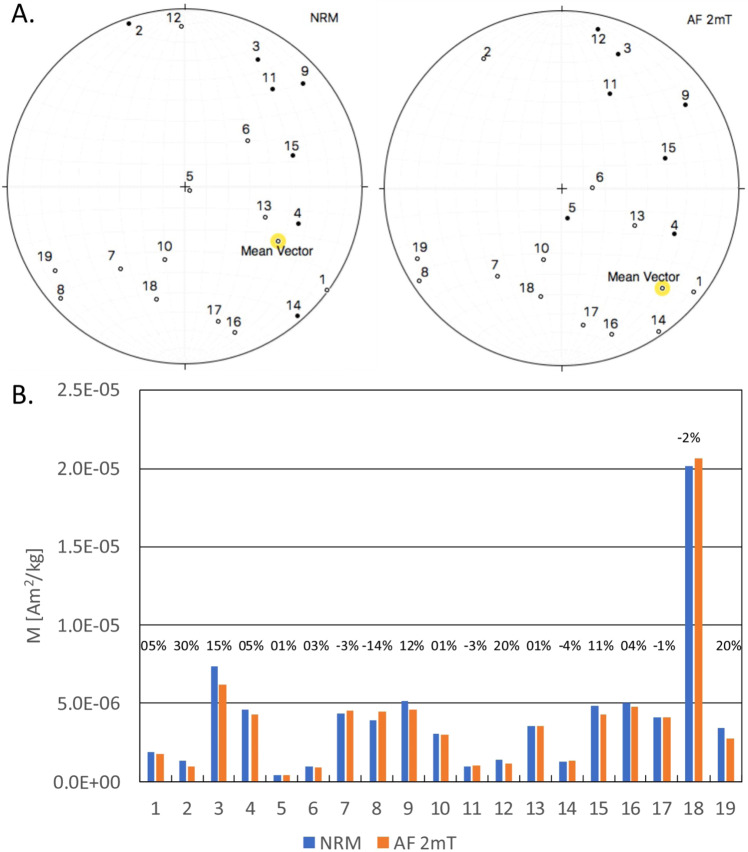
Directions and intensities of the oriented spheroids compared before and after cleaning with 0.002 T alternating magnetic field. Fisher Mean Vectors for both NRM and AF 2 mT data are marked in the equal area stereonets by yellow circle. (**A**) Equal Area Stereonet projections for NRM and AF 2 mT data sets. (**B**) Remanent magnetization of the spheroid samples before and after cleaning with an alternating demagnetizing field of 2 mT. The degree of remanence lost or gain is indicated by percentages above the respected columns.

Nineteen (19) oriented Pook’s Pebble bed carbonate spheroids were collected from the outcrop in Central Belize and analyzed for direction and intensity of NRM (Fig. 4). Remanence directions spread over the large directional angle exceeding 180 degree. Conversely, preferred direction was upward towards south east, while no directional data were observed in north west direction. The directional pattern was more or less the same after magnetic cleaning by 2 mT alternating magnetic field to reduce the viscous magnetization effects. The Pook’s Pebble Bed spheroids had two orders of magnitude variability in magnetization intensity (from 0.03 to 2.00 × 10^−5^ Am^2^/kg), which did not change significantly after cleaning with the 2 mT alternating field (Fig. 4).

**Figure 5:**
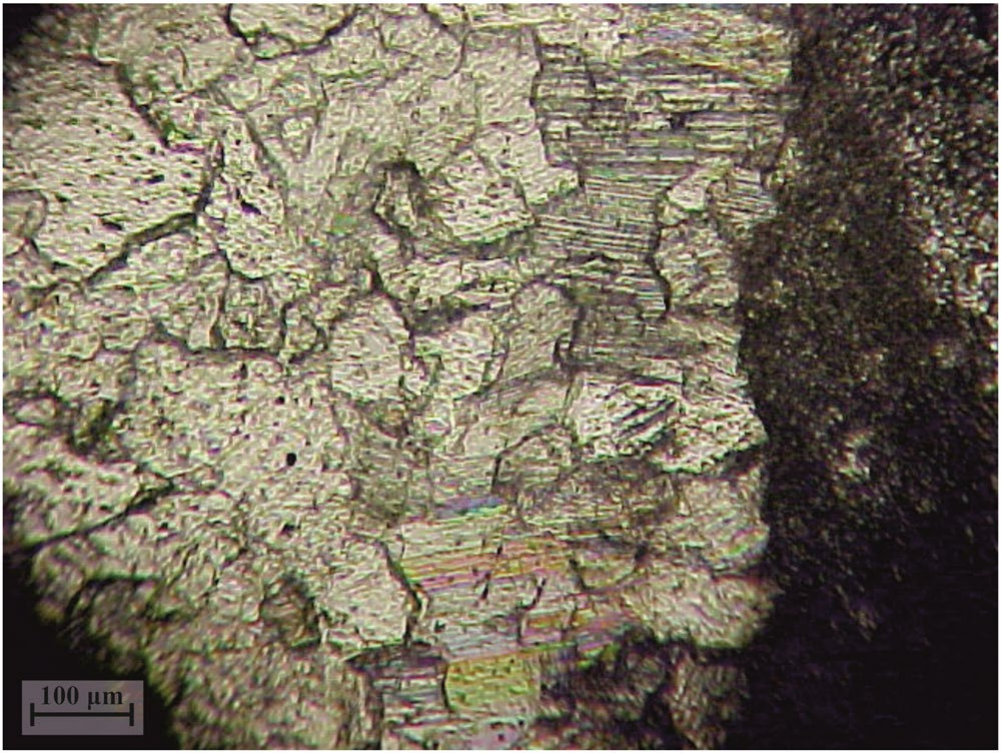
Micrograph (by G. Kletetschka) shows high orders of interference colors of calcite twinning along the standard 30 µm thick thin section cut from the profile of the Pook’s carbonate pebble. Image shows the interior of the pebble in the left part of the figure and pebble’s surface identified as a rapid change from the twinned calcite crystals in to the dark color (epoxy resin).

Several geologic thin sections were made from the carbonate pebbles and cobbles from the Albion Formation Pook’s Pebbles Bed. The thin sections were examined under the cross polarized light and contained coarse grained minerals. High order of interreference colors confirmed the prevalence of calcite grains. Interestingly, these crystals contained clear twinning along the surface of the pebble (Fig. 5), down to 0.2 mm depth after which the calcite twinning disappears, perhaps due to the high temperature annealing that the Pook’s Pebbles were exposed during the impact process.

**Figure 6:**
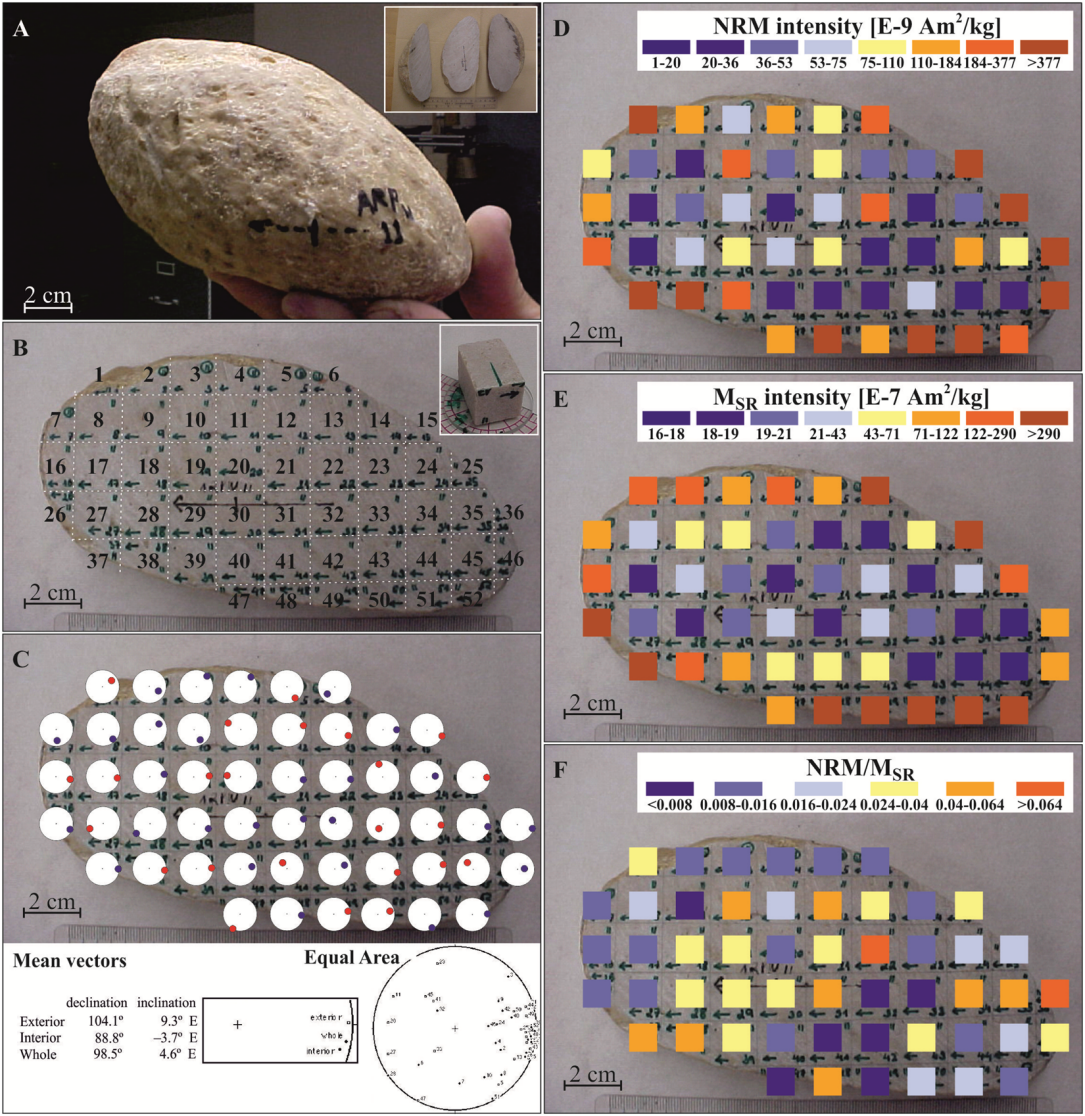
Magnetic properties of the Pook’s Pebble samples: (**A**) Photo of the pebble before it was cut in three slices (see small inset figure showing three slices). The photo shows how the pebble (labelled as ARP11) was oriented in space at the time of collection. The arrow is a trend direction (11 degree from the north) that resulted from intersection of the horizontal plane and pebble. The perpendicular line across the trending arrow is in a vertical direction. The top of the pebble is up the page. (**B**) The longitudinal cut shows description labels and green orientation arrows that are consistent with the strike and dip measurement (N11E, 90). Inset figure shows one of the subsample cubes. (**C**) Individual magnetic directions for subsamples are shown in term of miniature stereo net, where blue indicate down, into the page, direction and red up, from the page, directions. Inset at the bottom shows statistical analysis of the paleomagnetic directions of exterior and interior material along with the stereonet with all directions plotted. (**D**) NRM intensities are indicated by color scale E. Magnetization of Saturation Remanence (Msr) of subsamples is indicated by color scale shown. F. Efficiency of remanence acquisition (NRM/Msr) for the sub-fragments for Pook’s pebble are indicated by color scale shown. Photos by G. Kletetschka.

The interior slice cut from 12 cm carbonate Pook’s Pebble for magnetic analysis, showed white interior while the surface was distinctly pink (Fig. 6A). 52 subsamples (mostly cubes, except the surface containing sub samples) made from the interior slice varied in magnetization intensity from its minimum value 1.58 10^−9^ A m^2^/kg to its maximum 2.02 10^−6^ A m^2^/kg. While the average value was 1.68 10^−7^ A m^2^/kg, the median was 7.96 10^−8^ A m^2^/kg. Specimens containing the surface material were distinctly more magnetic and varied in magnetization intensity from its minimum value 4.49 10^−8^ A m^2^/kg to its maximum 2.02 10^−6^ A m^2^/kg. While the average value is 2.90 10^−7^ A m^2^/kg, the median was 1.75 10^−7^ A m^2^/kg. These parameters are all higher than the cubes from the inside that varied in magnetization intensity from its minimum value 1.58 10^−9^ A m^2^/kg to its maximum 2.05 10^−7^ A m^2^/kg. While the average value was 5.50 10^−8^ A m^2^/kg, the median was 4.38 10^−8^ A m^2^/kg.; see also the coloration scale in Fig. 6D).

After giving the cubes saturation magnetization inside 2 T magnetic field, the Msr intensity showed similar enhancement (Fig. 6E), with distinctly higher magnetization near the edges where it varied in magnetization intensity from its minimum value 4.27 10^−6^ A m^2^/kg to its maximum 3.57 10^−5^ A m^2^/kg. While the average value was 1.36 10^−5^ A m^2^/kg, the median was 9.93 10^−6^ A m^2^/kg. Specimens containing the interior material were distinctly less magnetic and varied in magnetization intensity from its minimum value 1.62 10^−6^ A m^2^/kg to its maximum 4.23 10^−6^ A m^2^/kg. While the average value of latter was 2.23 10^−6^ A m^2^/kg, the median was 2.02 10^−6^ A m^2^/kg. These interior parameters are all lower than the cubes from the outside (see also the coloration scale in Fig. 6E). While the NRM levels of the interior are not statistically significant due to larger noise level, the Msr values of the exterior sub-fragments of the pebble (average Msr = (1.36 +/−0.85) 10^−5^ A m^2^/kg) are significantly larger than Msr level of the interior (average Msr = (2.23 +/−0.70) 10^−6^ A m^2^/kg). See Tables S1, S2.

Recent theoretical analysis showed that ratio between the NRM and Msr intensities follows the simple linear TRM acquisition that indicates the paleofield at which the rock was cooled and acquired its TRM^16^. In most of the samples that originate in terrestrial conditions with geomagnetic field, such ratio is near 0.02^17^. Even though the Pook’s Pebble Bed samples most likely did not reach temperatures high enough to acquire strictly TRM for all of the samples, the ratio (Fig. 6, Table S1, S2) shows spread of values between 0.001 and 0.1. The minimum and maximum values of the NRM/Msr ratio (Fig. 6F) are distributed through the pebble and form distinct clusters. The top of the Pook’s Pebbles samples did not contain any of the extreme values, while the bottom only contained 3 areas with such values (Fig. 6F).

**Figure 7:**
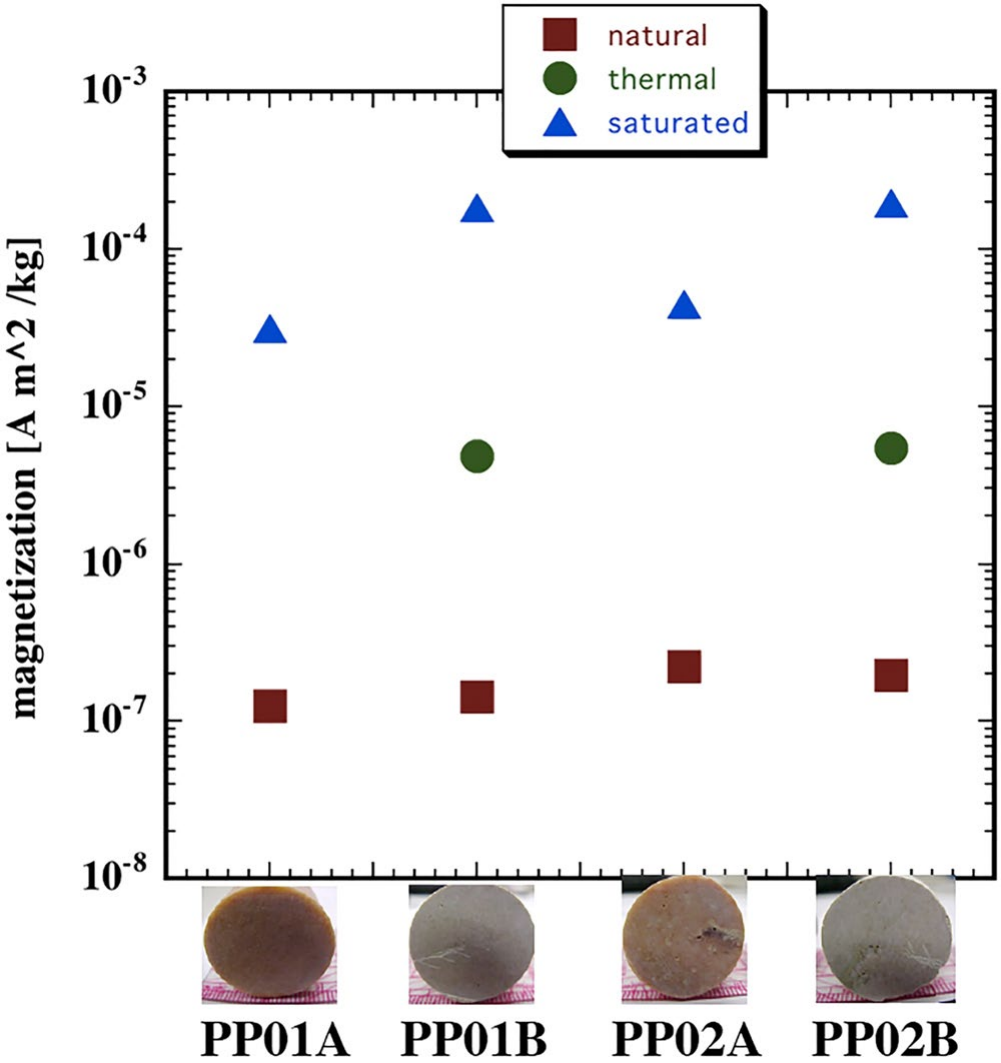
Ranges of natural remanent, thermal, and saturated magnetization on two, PP01, PP02, 1 inch in diameter, cylindrical pair samples (pair PP01A, PP01B and pair PP02A, PP02B) of Pooks Pebble samples. Photos by G. Kletetschka.

In order to find out the values of TRM for Pook’s Pebble material, we compared NRM, TRM and Msr for two representative sample pairs of similar Pook’s Pebble material (Fig. S5). Figure 7 indicates that while NRM for these two samples is near 100 × 10^−9^ Am^2^/kg, TRM values acquired at 30 µT were about 5,000 × 10^−9^ Am^2^/kg and Msr values were over 100,000 × 10^−9^ Am^2^/kg. Values were near 30,000 × 10^−9^ Am^2^/kg for non-heated samples (Fig. 7).

## Discussion

The Pook’s Pebbles Bed samples morphology resembles meteorites by showing an aerodynamically deformed crust with well-rounded surfaces, facets, crusts, and pits (Figs. S3–S7). For example, iron meteorites pits are places where ablation and heating by the air removed preferentially iron sulfide impurities resembles the deeper pitting in Pook’s Pebbles. The Pook’s Pebbles have commonly high occurrence of pitting on a single side suggesting that this could form by ablation in the high-speed dust environment that followed the impact. The surface of Pook’s Pebbles displays several elements in common with fusion crusts on meteorites. The carbonate minerals contain twinning recrystallization which may be due to pressure/temperature modification during the pebbles’ ablation. The calcite twinning down to 0.2 mm depth below the surface of Pook’s Pebble (Fig. 5) suggests that the pebbles experienced impact pressures between 100 and 500 MPa^18^. This observation indicates that some of the Pook’s Pebbles may have experienced shock pressure commonly observed in carbonaceous meteorites^18^. Meteorites may contain vesicular fusion crusts. These vesicular flows resemble the layered crusts with truncations and with vugs and striations found in the Pook’s Pebbles (Fig. S6). Ablation and heating during atmospheric acceleration in the highly particle rich environment, possibly, when pebbles were ejected and airborne, could locally melt the surface (Fig. S1) due to the atmospheric drag as it travels in the vapor plume cloud and form these features. Also, collision between Pook’s pebbles, of these “Chicxulub meteorite-like” carbonates, could have caused a lot of the demarcation found in their surface. The Albion Formation Pook’s Pebble Bed contains pebbles and cobbles with polished surfaces, striation marks (Fig. S6), and gouges-like indentations (Fig. S7). Somewhat similar morphologies have been reported from the ejecta blanket of the Ries crater in Germany^19^. These surface modifications are superimposed on the preexisting ablation features and may have formed by particle interactions and collisions as the ejecta passed through a near-surface.

One of the spheroid bed components, pink spheroids, showed slightly enhanced magnetization compared with limestone fragments and white spheroids. Recent study^20^ showed that the pink colored spheroids contain more iron hematite nanoparticles than white spheroid containing goethite. This is consistent with our finding as presented in Fig. 3D. While white spheroids and limestone fragments are diamagnetic, showing negative values, when exposed to 2 T external field, the pink spheroid’s hematite content^20^ distinguishes the pink spheroid material from being diamagnetic.

Spheroids in our study indicate two orders of magnitude spread of magnetization values (Fig. 4) and might be consistent with the observation that spheroids may contain either hematite or goethite^20^, two minerals with similar magnetic properties, except for saturation magnetic remanence which is larger for hematite^16^.

As an observation, Pook’s pebbles with larger magnetic intensity may contain larger amount of hematite. Our data (Fig. 6A,B) shows that while the surface of the Pook’s Pebble is distinctly pink the interior has a whitish gray color. This may imply that the core of the Pook’s Pebbles may have being less modified than the crust of the pebbles by Chicxulub impact process. Both NRM and Msr revealed that the surface of the Pook’s Pebbles specimens is magnetically enhanced. We interpret this observation as the Pook’s Pebble was exposed to high temperature dusty nanoparticles of limestone/dolomite debris from the vapor plume cloud. When dust experienced larger temperatures, the iron hydroxide (goethite) containing carbonate reduced to hematite with larger magnetization. This interpretation is supported by our experimental heating of fragments of the Pook’s Pebbles (Fig. 7). Note that heating increased Msr by an order of magnitude. This experiment confirmed that when the limestone/dolostone material is heated, the iron content in form of goethite inside the pebble, changes into more magnetic phase, hematite^20^.

Magnetic properties of Spheroid Bed material separated its constituents based on their saturation magnetization. Data revealed significant magnetic enhancement of the devitrified glass compared to the other three components, pink and white spheroids and limestone fragments (Fig. 3D). The devitrified glass has substantial paramagnetic component as a result of presence of clay that has been devitrified from impact glass^21^. Clay content has been known to have significant paramagnetic nature^22^. In this work, devitrified glass from Chicxulub can be then distinguished using magnetic sensing techniques. This new finding can be utilized in future planetary robotic missions to detect of anomalous paramagnetism, which could lead to identification of impact glass on planetary surfaces^23^.

Paleomagnetism of nineteen oriented spheroids (Fig. 4A) showed significant spread of the directions in respect to the reversed magnetic field that was present at the time of impact^14,15^. At the time of spheroid deposition, the field was reversed, consistent with the polarity chron 29 R at the time of Chicxulub impact event^23,24^. Note that the directions of magnetization seem to spread around axis dipping steeply toward the south east direction. This observation suggests two ways of acquisition of the observed magnetic remanence. First possibility assumes that the Pook’s Pebbles were dropping down from the hot atmosphere and due to aerodynamic shape of the pebbles they acquired magnetic component during this fall. Assuming the Pook’s Pebbles were falling more or less vertically, during the Chicxulub impact deposition process, the pebbles preserved the vertical orientation while they moved slightly in horizontal plane and caused the directional spread. The second acquisition assumes that pebbles were still quite hot at the time of landing on the ground and thus they acquired their magnetization *in situ*. Pebbles often contained inside a fragment of limestone or dolomite that formed accretionary lapilli with the clay around it and formed spheroids. This small fragment may have kept its prior magnetic remanence that was in random orientation after landing. The thermal overprint during the cooling would result in directional scatter that is consistent with the large spread of directions around the reversed magnetic field. Therefore, while the overall magnetic direction is consistent with the direction of the applied magnetic field at the time of the Chicxulub impact, the directional spread may be either the evidence of aerodynamic landing and/or the result of sum of magnetic vector acquired after the landing and the magnetization vector of the cold interior with preexisting magnetization.

Note that the magnetization of the Pook’s Pebble in Fig. 6C is antiparallel to the green arrows on individual subsamples (Fig. 6B) that points in the direction 10 degrees east from north, the direction marked on the pebble at the collection time (Fig. 6A). This observation indicates that the overall pebble’s magnetization is in reversed direction to the todays magnetic field, consistent with the magnetization of the spheroids (Fig. 4).

Additionally, Fig. 6D suggests that variation of NRM has larger variation inside the Pook’s Pebble than variation of Msr of the same subsamples. After adoption of the level of magnetization indicator NRM/Msr^16^ we noted that the level of magnetization values were spread over 2 orders of magnitude. The ratio between the TRM and saturation remanence (Msr) relates to the magnitude of the geomagnetic field that magnetized the rock^16,25^. Because the geomagnetic field during its history is generated by geodynamo, the magnitude of the flux on the Earth’s surface stays within tens of microtesla. Such flux intensity magnetizes terrestrial rocks during their cooling renders the acquisition of TRM to about 2% of its saturation magnetization, see Kletetschka and Wieczorek (2017)^16^ and related equations. This value was used as our choice to split the NRM/Msr values in Fig. 6F. Values larger than 0.024 are in shades of yellow and red, while lower values are in shades of blue. When plotting these extreme values over the Pook’s Pebble slab, we note that the pebble contains clusters with larger level of magnetization than common geomagnetic field values next to material with lower level of magnetization values than geomagnetic field (Fig. 6F). The direction of magnetization also greatly changes from one sample to the another (Fig. 6C). The directional and magnitude changes in magnetization suggest that the Pook’s Pebble experienced contrasting magnetic acquisition on small scale, consistent with an electric discharge^26^. The non-homogeneous magnetic fields of electric currents could have therefore magnetized portions of the material while other material was demagnetized. Electric current magnetizes preferably low coercivity grains as this happen in low temperature^26^. Evidence of magnetization by electric current is supported by analyzing the alternating field demagnetization spectra (Fig. S8) where we see that field required to demagnetize NRM to less than 50% of its original NRM is about order of magnitude lower than field required to demagnetize the Msr. Such observation is consistent with the magnetizing by electric discharge because the carbonate material is not heated during this process and essentially acquires isothermal remanent magnetization^26^.

## Conclusions

Given that the Chicxulub Albion Formation Pook’s Pebbles’ surface features are superimposed on the ablation features, we propose that they were formed by particle interactions and collisions as the ejecta passed through a near-surface debris cloud and struck the regolith-covered Cretaceous land surface. Required ejection velocities of 1–2 km/s to reach Belize produce trajectories that that reach altitudes of approximately 100 km. Twinning along the Pook’s Pebbles’ surfaces indicates that some of the them experienced shock pressure up to 500 MPa. We found that devitrified impact glass has distinct paramagnetic enhancement. This property can be utilized as an indicator for future planetary robotic missions, where detection of anomalous paramagnetism can lead to identification of impact glass on the planetary surfaces^23^. Furthermore, we showed that the pink spheroid’s hematite content^20^ distinguishes this material from being diamagnetic or tending to become magnetized in a direction at 180 degrees to the existing magnetic field. While the overall magnetic direction is consistent with the direction of the applied magnetic field at the time of the Chicxulub impact the directional spread may be either the evidence of aerodynamic landing and/or the result of the sum of a magnetic vector acquired after the landing and the magnetization vector of the cold interior with preexisting magnetization. Our magnetic analyses reveal that pebbles vary in magnetic intensity due to larger amount of hematite. Furthermore, reddening of the Pook’s Pebbles’ surfaces is associated with larger magnetizations. And, due to larger temperatures, the iron hydroxide (goethite)-containing dust reduced to hematite with large magnetization potential. The magnetic characterization of the Albion Formation Pook’s Pebble revealed that the directional and magnitude changes in magnetization of sub-fragments of Pook’s Pebbles is consistent with exposure to electric currents that magnetized/demagnetized portions of the material with a non-homogeneous magnetic field, which may provide insights into the debris cloud environment that this impact material was exposed to. Furthermore, the magnetization signature of the Albion Formation Spheroid Bed, show that this technique could be used as a signature for impact ejecta deposits detection by planetary rovers, and an instrument to be capable to make this type of measurement could enhance the payload for future planetary missions.

## Supplementary information

Supplementary information.
