# Supplementary material for: Electric discharge evidence found in a new class of material in the Chicxulub ejecta
Source: Sci Rep. 2020 Jun 3;10:9035. doi: 10.1038/s41598-020-65974-2 (PMC7271149; doi:10.1038/s41598-020-65974-2)
Supplement: Supplementary file 1 — Supplementary information. [file 41598_2020_65974_MOESM1_ESM.pdf]

Electric discharge evidence found in a new class of material in the Chicxulub ejecta

Supplementary Information

Gunther Kletetschka[1,2,3], Adriana Ocampo[4], Vojtech Zila[5], and Tiiu Elbra[1]

1. Geological Institute, Czech Academy of Sciences, 165 00 Prague 6, Rozvojová 269; Czech Republic;
2. Department of Applied Geophysics, Charles University, Albertov 6, 120 00 Prague 2, Czech Republic;
3. Geophysical Institute, University of Alaska, Fairbanks, 903 N Koyukuk Drive, Fairbanks, AK, USA,
4. NASA Headquarters, Washington DC 20546, USA
5. Department of Infectious Diseases, Virology, University of Heidelberg, Germany

Corresponding author: Gunther Kletetschka, kletetsg@natur.cuni.cz

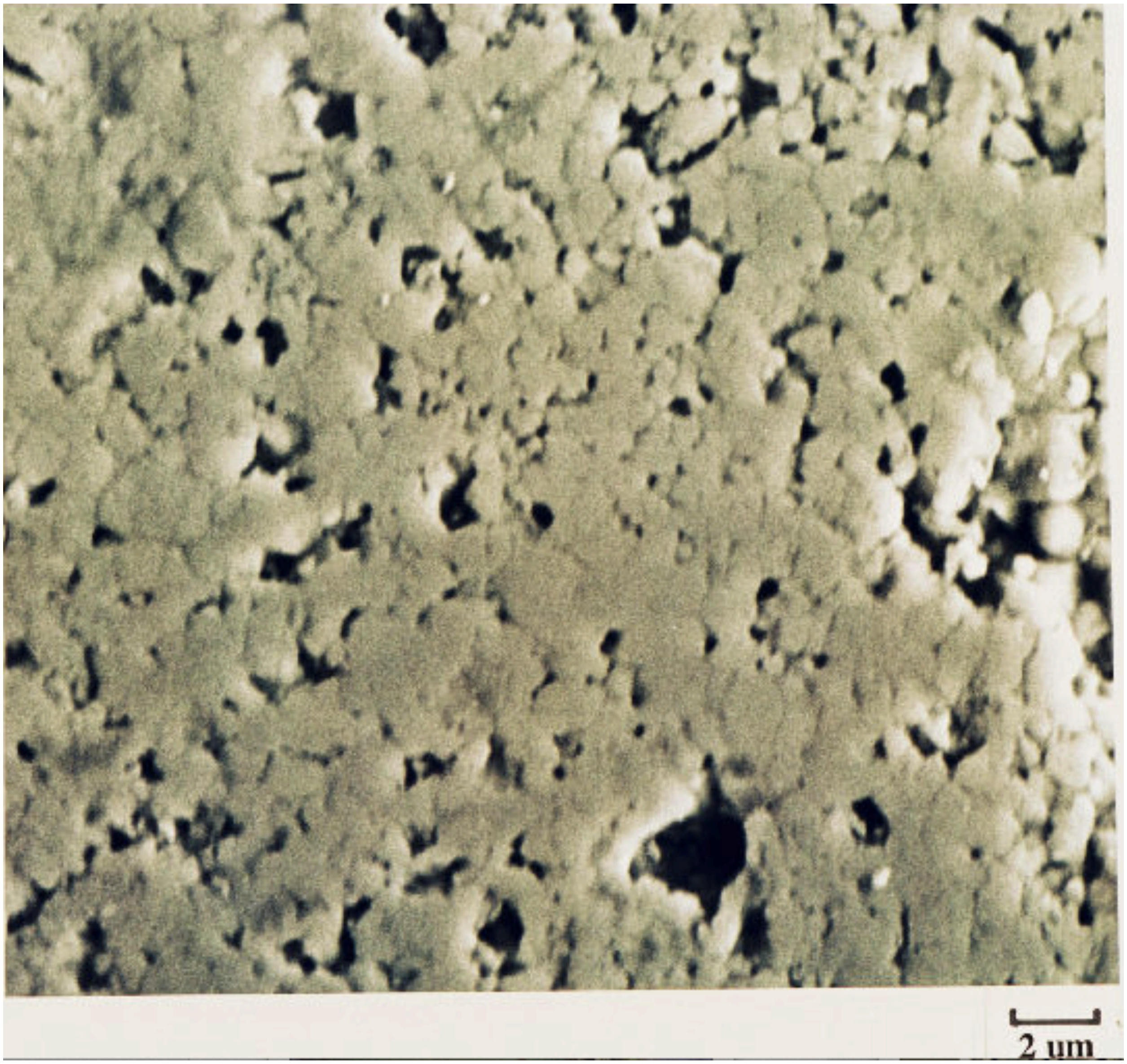

Figure S1: Scanning Electron image of the fuzzed calcite grains on the surface of the Albion Formation's Pook's Pebble.

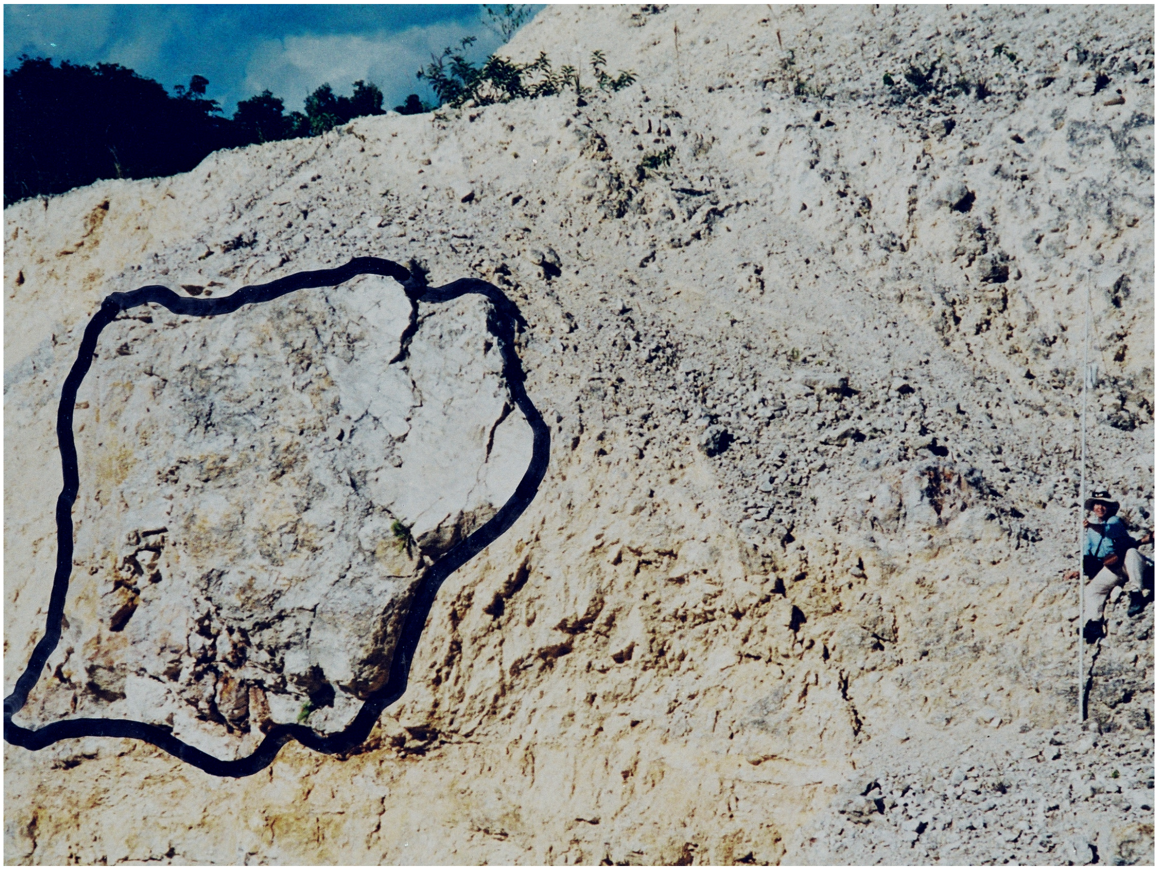

Figure S2: Field photo of the clast that exceeds 5 m in its size. Adriana Ocampo is at the right part hold 5 m long stick for scale

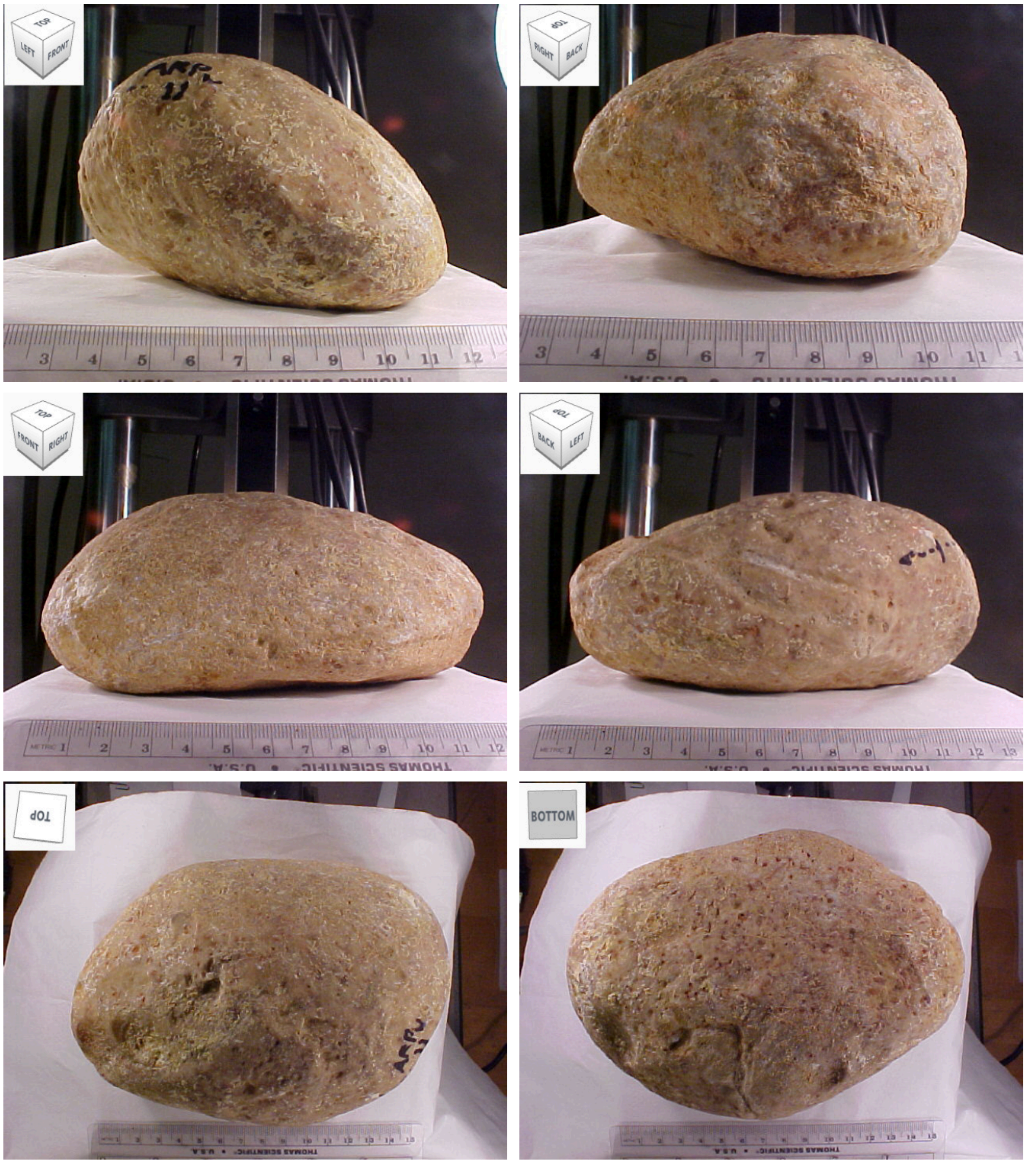

Figure S3: Pebble ARP11 before cutting is shown from six different angles. The specific view is indicated by the cube in upper left corner of each image.

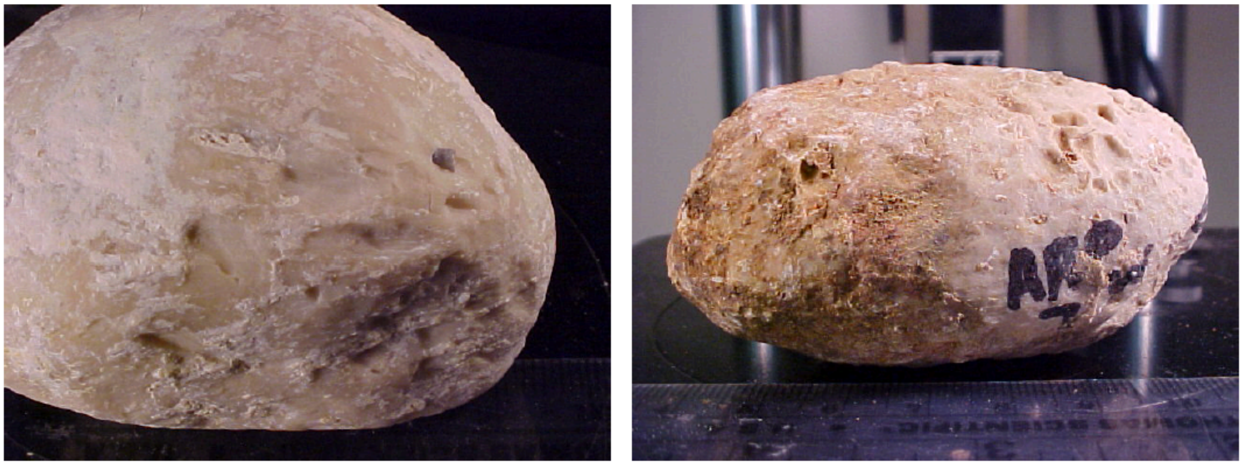

Figure S4: Examples of the Pook's Pebble's striations' morphology. Left and right pebbles have 7 cm and 6 cm, respectively.

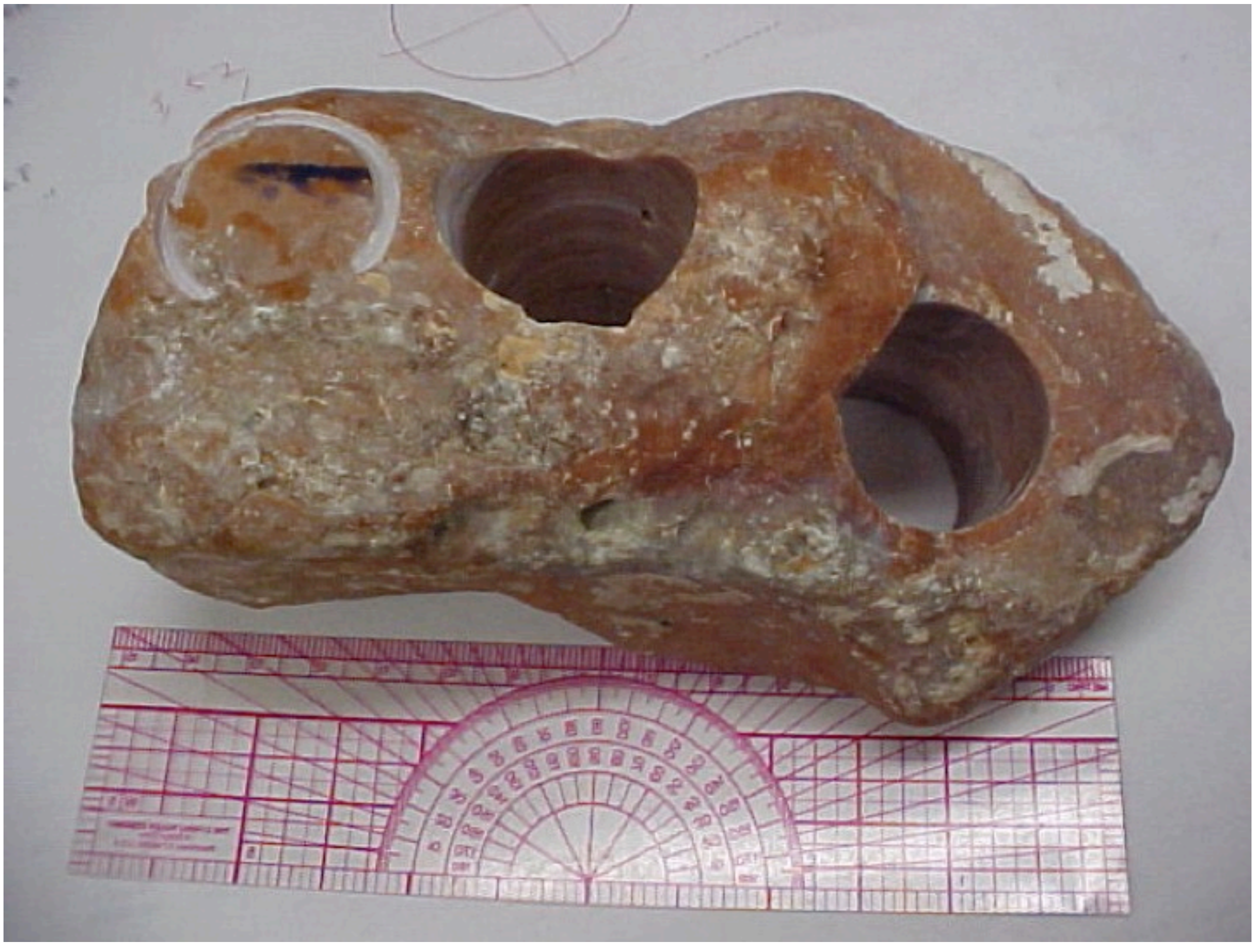

Figure S5: Pook's Pebble 04 that was used for collection of cylindrical samples for rock magnetic measurements.

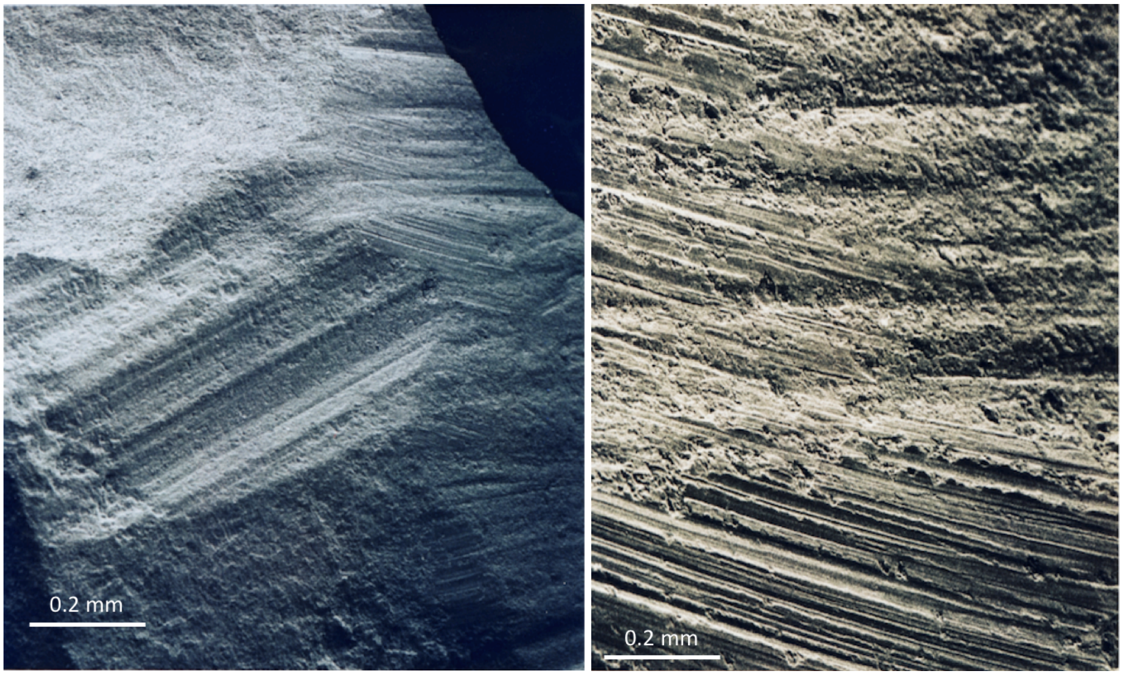

Figure S6: Examples of striations and grooves on the surfaces of Pook's Pebbles collected.

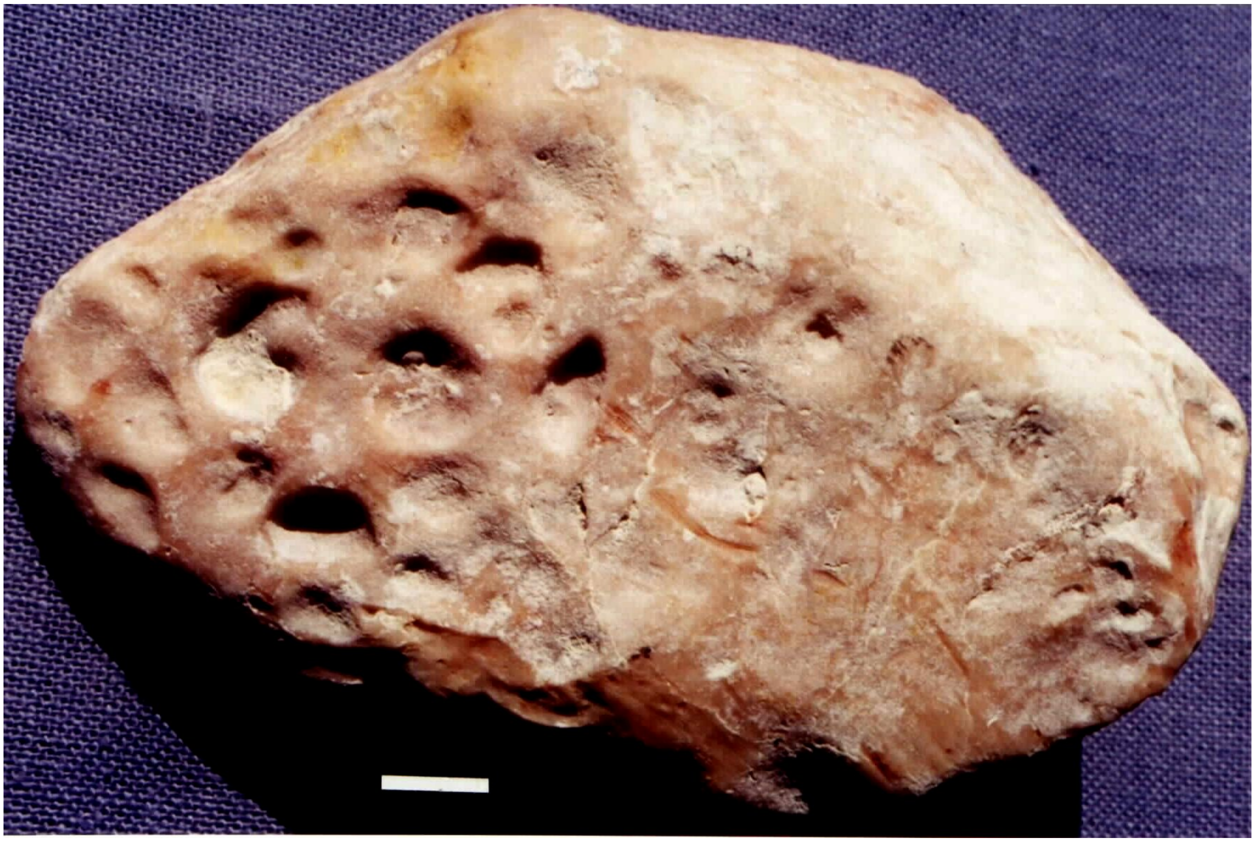

Figure S7: Sample of Pook's Pebble (07) with rounded indentation. The white bar is one (1) cm. Image by Adriana Ocampo.

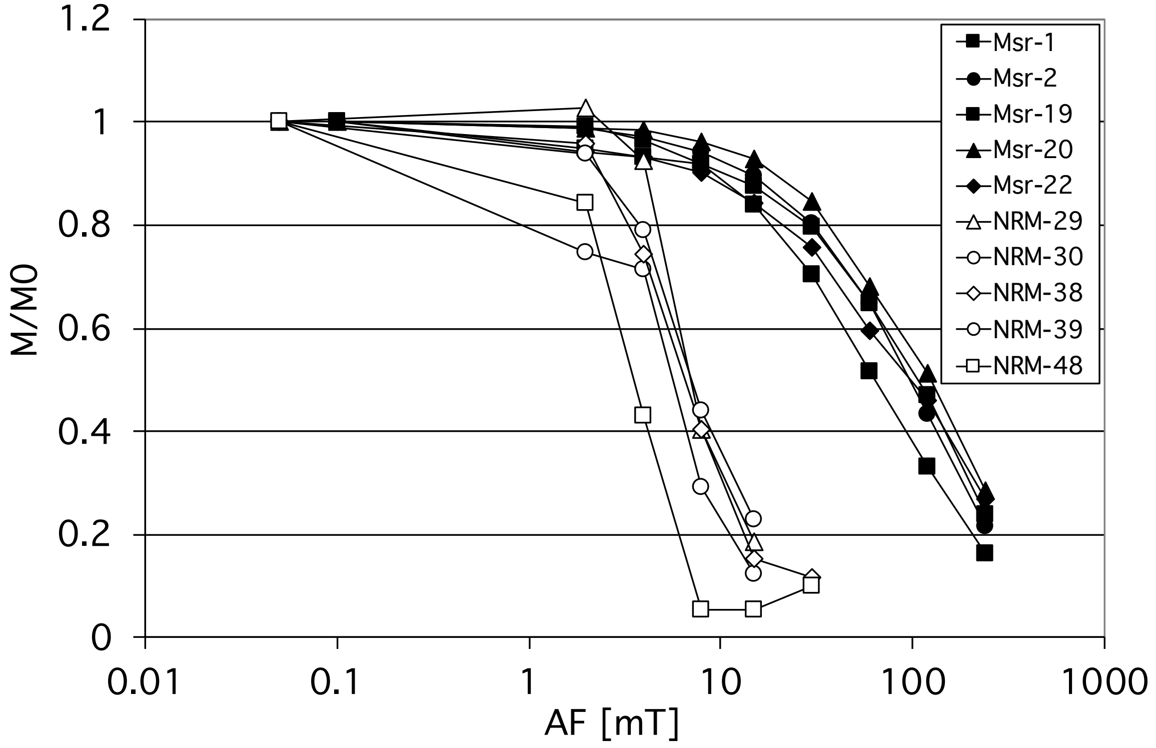

Figure S8: Examples of alternating field demagnetization of NRM and Msr of the Pook's pebble samples.

Table S1: Exterior samples' values of the natural remanent magnetizations (NRM) and saturation remanence (Msr) and the ratio REM between "NRM" and "Msr". Statistical parameters show average values, standard deviations, medians, minimum (min) and maximum (max) values.

exterior			
**sample**	**NRM** [Am^2/kg]	Msr [Am^2/kg]	**REM**
1	2.61E-07	9.50E-06	2.75E-02
2	1.16E-07	9.18E-06	1.26E-02
3	6.24E-08	5.28E-06	1.18E-02
4	1.34E-07	9.42E-06	1.42E-02
5	7.84E-08	7.05E-06	1.11E-02
6	1.84E-07	1.43E-05	1.29E-02
7	8.27E-08	6.32E-06	1.31E-02
14	4.49E-08	4.27E-06	1.05E-02
15	3.65E-07	1.41E-05	2.59E-02
16	1.30E-07	9.93E-06	1.31E-02
25	2.56E-07	1.22E-05	2.10E-02
26	1.55E-07	1.49E-05	1.04E-02
36	3.94E-07	5.87E-06	6.72E-02
37	7.15E-07	1.71E-05	4.19E-02
38	5.02E-07	8.40E-06	5.97E-02
39	1.75E-07	5.19E-06	3.37E-02
46	2.41E-07	6.40E-06	3.77E-02
47	1.30E-07	2.90E-05	4.47E-03
48	2.02E-06	3.57E-05	5.66E-02
49	1.05E-07	2.21E-05	4.76E-03
50	3.77E-07	1.90E-05	1.99E-02
51	3.46E-07	1.98E-05	1.75E-02
52	2.33E-07	2.73E-05	8.55E-03
average:	3.09E-07	1.36E-05	2.33E-02
std=	4.06E-07	8.52E-06	1.80E-02
median=	1.84E-07	9.93E-06	1.42E-02
min=	4.49E-08	4.27E-06	4.47E-03
max=	2.02E-06	3.57E-05	6.72E-02

Table S2: Interior samples' values of the natural remanent magnetizations (NRM) and saturation remanence (Msr) and the ratio REM between "NRM" and "Msr". Statistical parameters show average values, standard deviations, medians, minimum (min) and maximum (max) values.

interior			
**sample**	**NRM** [Am^2/kg]	**Msr** [Am^2/kg]	**REM**
8	4.38E-08	2.50E-06	1.75E-02
9	2.42E-08	3.03E-06	7.99E-03
10	1.74E-07	2.90E-06	6.01E-02
11	3.86E-08	2.02E-06	1.91E-02
12	8.09E-08	1.76E-06	4.59E-02
13	5.23E-08	1.62E-06	3.23E-02
17	2.70E-08	1.88E-06	1.44E-02
18	5.06E-08	2.10E-06	2.42E-02
19	7.45E-08	2.07E-06	3.60E-02
20	1.49E-08	1.86E-06	8.01E-03
21	7.17E-08	2.04E-06	3.51E-02
22	2.05E-07	2.29E-06	8.93E-02
23	1.74E-08	1.68E-06	1.04E-02
24	5.29E-08	2.22E-06	2.38E-02
27	1.98E-08	2.04E-06	9.68E-03
28	5.74E-08	1.90E-06	3.02E-02
29	7.49E-08	1.98E-06	3.78E-02
30	5.34E-08	2.11E-06	2.54E-02
31	8.11E-08	1.82E-06	4.46E-02
32	1.58E-09	2.27E-06	6.97E-04
33	1.14E-08	1.98E-06	5.74E-03
34	1.10E-07	1.85E-06	5.91E-02
35	9.96E-08	1.71E-06	5.82E-02
40	3.61E-08	4.09E-06	8.82E-03
41	2.46E-08	3.66E-06	6.72E-03
42	2.44E-08	4.23E-06	5.78E-03
43	6.66E-08	1.68E-06	3.96E-02
44	1.45E-08	1.67E-06	8.66E-03
45	3.54E-08	1.76E-06	2.02E-02
average:	5.65E-08	2.23E-06	2.71E-02
std=	4.61E-08	6.98E-07	2.11E-02
median=	5.06E-08	2.02E-06	2.38E-02
min=	1.58E-09	1.62E-06	6.97E-04
max=	2.05E-07	4.23E-06	8.93E-02
